# Priorities for research and control of cestode zoonoses in Asia

**DOI:** 10.1186/2049-9957-2-16

**Published:** 2013-08-01

**Authors:** Ning Xiao, Jia-Wen Yao, Wei Ding, Patrick Giraudoux, Philip S Craig, Akira Ito

**Affiliations:** 1 National Institute of Parasitic Diseases, Chinese Center for Disease Control and Prevention; WHO Collaborative Center for Malaria, Schistosomiasis and Filariasis; Key Laboratory of Parasite and Vector Biology, Ministry of Health, Shanghai 200025, People’s Republic of China; 2 Department of Chrono-Environment, University of Franche-Comté/CNRS and Institut Universitaire de France, Besançon, France; 3 Cestode Zoonoses Research Group, School of Environment and Life Sciences, University of Salford, Manchester UK; 4 Department of Parasitology, Asahikawa Medical University, Asahikawa 078-8510, Japan

**Keywords:** Neglected diseases, Cestode zoonoses, Asia, Priority, Gap, Research, Control

## Abstract

Globally, cestode zoonoses cause serious public health problems, particularly in Asia. Among all neglected zoonotic diseases, cestode zoonoses account for over 75% of global disability adjusted life years (DALYs) lost. An international symposium on cestode zoonoses research and control was held in Shanghai, China between 28^th^ and 30^th^ October 2012 in order to establish joint efforts to study and research effective approaches to control these zoonoses. It brought together 96 scientists from the Asian region and beyond to exchange ideas, report on progress, make a gap analysis, and distill prioritizing settings with a focus on the Asian region. Key objectives of this international symposium were to agree on solutions to accelerate progress towards decreasing transmission, and human mortality and morbidity caused by the three major cestode zoonoses (cystic echinococcosis, alveolar echinococcosis, and cysticercosis); to critically assess the potential to control these diseases; to establish a research and validation agenda on existing and new approaches; and to report on novel tools for the study and control of cestode zoonoses.

## Multilingual abstracts

Please see Additional file 1 for translations of the abstract into the six official working languages of the United Nations.

## Review

### Background

Cestode zoonoses are emerging, re-emerging or spreading worldwide, and are classed as neglected infectious diseases or neglected tropical diseases (NTDs), and neglected zoonotic diseases (NZDs) [1,2]. Among all NZDs, Asia currently has the greatest burden of cystic echinococcosis (CE) and alveolar echinococcosis (AE) in humans with these accounting for over 75% of global disability adjusted life years (DALYs) lost, and primarily occurring in west and north China and the Central Asian Republics [3-7]. Many joint efforts have been made to reduce or even eliminate the transmission of this group of zoonoses [8]. Over the last decade or so, two important symposia on cestode zoonoses were organized in China (in 2000 and 2006), and one workshop in Japan in 2011 [9-11]. In 2006, the Chinese Government launched a national control program for echinococcosis, the largest such program in the world [12,13]. However in Asia, many challenges still need to be overcome to effectively deal with these diseases. Challenges include the lack of adequate investment, and insufficient understanding of disease transmission at the community level and its molecular epidemiology. Applications of modern tools are required to obtain evidence about pathogenic aspects, nature of genotypes or isolates, as well as specific confirmation/diagnosis of the diseases themselves. Further understanding the epidemiology and the transmission ecology at different scales is also required. Furthermore, we should identify research gaps and optimize strategies for control or even elimination of cestode zoonoses in specified region(s).

To respond to the increased requirement for strengthening international collaborations on research and control of cestode zoonoses with a focus on the Asian region, an international symposium was jointly proposed and co-organized in Shanghai by the National Institute of Parasitic Diseases, part of the Chinese Center for Disease Control and Prevention, and the Asahikawa Medical University in Japan. A total of 96 scientists attended and engaged in topics as diverse as treatment, diagnosis, molecular biology, epidemiology, transmission ecology, public health and health policy, and progress in research and control of cestode zoonoses in Asia, including gap analysis and priority settings.

### Methods

This international conference focused on the control of cestode zoonoses, and involved 96 participants from 11 countries, the Chinese Ministry of Health (MOH), the World Health Organization (WHO) Regional Office and non-government organizations (NGOs). During the two-day symposium, five keynote speakers introduced the current global situation of cestode zoonoses from various perspectives. Thirteen speakers addressed the Asian status of cestode zoonoses, and recent achievements on biological, spatial, molecular and diagnostic aspects. Twenty-two participants in four parallel sessions shared their experiences and progress on epidemiology, ecology, biology, immunology, molecular taxonomy and phylogeny, control strategy, diagnosis, vaccine research, clinical treatment, and public policy related to cestode zoonoses control (see Tables 1 and 2).

**Table 1:** The participants-affiliated institutions and their major research fields on echinococcosis

**Institutions**	**Major research fields**						**References**
	Epidemiology*	**Diagnosis**	**Control, treatment, and vaccine**	-Omics**	**Taxonomy**	Ecology***	
Asahikawa Med Univ., Japan.	☆	☆	☆		☆		Ito A, 2003, 2006, 2007; Xiao N, 2005, 2006; Knapp J, 2011; Mamuti W, 2006a,b; Nakao M, 2007, 2010; Sako Y, 2009.
East China Normal Univ., China.						☆	Jiang W, 2012
Fudan Univ., China.				☆			Cui SJ, 2013
Hokkaido Univ., Japan.			☆				Dang ZS, 2012
Inst. of Parasit Dis, Sichuan CDC, China.	☆		☆				Li TY, 2005; Huang Y, 2012; Xiao N, 2012.
Inst. of Systematics and Ecology of Animals, Russia.	☆					☆	Konyaev SV, 2012a,b
National Inst. of Parasit Dis, China CDC.	☆	☆	☆	☆			Wang LY, 2010; Liu CS, 2012; Wang Y, 2012; Xiao N, 2012.
Ningxia Med Univ., China.						☆	Yang YR, 2010
Universita di Pavia, Italy.		☆	☆				Brunetti E, 2004, 2010.
Univ. of Franche-Comte, France.	☆					☆	Giraudoux P, 2006, 2013; Pleydell DR, 2008.
Univ. of Salford, UK.	☆						Craig PS, 1991, 2000, 2006.
Univ. of Ulm, Germany.		☆	☆				Kern P, 2006
Univ. of Zurich, Switzerland.	☆						Budke CM, 2004; Torgerson PR, 2006.
Xiamen Univ., China.	☆				☆		Tang CT, 2006
Xinjiang Med. Univ., China		☆	☆		☆		Bart JM, 2006; Abstract book of the symposium, 2012.

*Note:* The institutions are listed in alphabetical order and the references shown in the table are those that have been published.

*Epidemiology contains disease burden research.

**In this table, omics consist of genomics, proteomics, transcriptomics, immunomics, etc..

*** Ecology includes animal epidemiology and identification of *Echinococcus* spp.

**Table 2:** The participants-affiliated institutions and their major research fields on taeniasis/cysticercosis

**Institutions**	**Major research fields**				**References**
	**Epidemiology**	**Diagnosis**	**Control and treatment**	**Taxonomy**	
Asahikawa Med. Univ., Japan	☆	☆	☆	☆	Ito A, 2003, 2006; Sako Y, 2009; Yanagida T, 2010; Knapp J, 2011; Nakao M, 2010; Nkouawa A, 2011.
Inst. of Parasit Dis., Sichuan CDC, China.			☆		Li TY, 2006, 2012.
National Inst. of Infect. Dis., Japan.			☆		Yamasaki H, 2013
Tottori Univ., Japan.				☆	Yamane K, 2012
Universidade Federal do Tocantins, Brazil.	☆			☆	Sato MO, 2011
Univ. of Ngaoundere, Cameroon.			☆		Assana E, 2013

*Note:* The institutions are listed in alphabetical order and the references shown in the table are those that have been published.

In the roundtable interaction, participants joined discussions about how to make a systematic assessment related to: a) the current endemic/epidemic situation of cestode zoonoses with a focus on Asian countries; b) the current national and international control strategies for cestode zoonoses; c) effective approaches to implement intersectoral efforts on survey and control of cestode zoonoses; d) importance of epidemic surveillance networks and information reporting systems; and e) opportunities, challenges, feasibilities, sustainability and technique demand for cestode zoonoses control.

During the symposium, the Delphi study was applied for obtaining and understanding control bottleneck, research and resource gaps, priority settings for research and control, and other related problems from all domestic and foreign participants [14]. Information was also collected through information exchange, roundtable discussions, questionnaires, and quantitative analysis.

### Progress on research and control for cestode zoonoses

The main contributions were in the following areas: biology and eco-epidemiology, control and interventions, molecular biology and diagnostics, gap analysis, and research priorities.

#### Biology and epidemiology

##### *Echinococcus*

According to recent surveys with a focus on epidemiology in wildlife, prevalences of *Echinococcus* spp were reported as high as 61.7-64.5% (*E. multilocularis* 19.2-48.4%, *E. shiquicus* 26.7-29.0%, and mixed infection of these two species 12.9-15.8%) in Tibetan fox populations [15,16]. A survey of *Echinococcus* spp in small mammals on the Tibetan plateau was also conducted by morphology and hemi-nested polymerase chain reaction (PCR) with a prevalence of 7.8%, in which 44 of 45 samples were identified by the hemi-nested PCR as infections of *E. multilocularis* (97.8%) and one considered as *E. granulosus* in *Microtus fuscus*[16]. Three morphologically different “species” observed at various stages were reported to be distributed sympatrically in the Hulunbeier pasture of Inner Mongolia, China, and was referred to as *E. multilocularis*, *E. sibiricensis* and *E. russicensis*[17]. These studies greatly influenced further studies on the genetic variation of *Echinococcus* species to be conducted*.* However, some of the above-mentioned lesions have been confirmed to be an intra-species variation of *E. multilocularis* by the most recent molecular studies [18,19]. The controversial data suggest that it is essential to carry out molecular identifications even further.

From continental to regional scales, human AE spatial distribution appears to be highly aggregated and forms discrete patches of endemicity within which hotspots of much larger prevalences may occur. Using regional spatial models helps to explain why transmission of *E. multilocularis* is more intense in distinct ecological systems with various intermediate host communities, landscape, and climates in continental Asia [20-25]. The dissolution of the Soviet Union was presented in the symposium, showing that such an event caused great socioeconomic changes that pushed people back into traditional small-holding livestock husbandry which resulted in both human CE and AE emerging or re-emerging [5,6]. Due to the long asymptomatic development of the parasite in humans, monitoring dog and/or fox populations is recommended as a better way to anticipate risks for human exposure [26].

The analysis of spatial patterns of human and animal case distribution can provide information on transmission processes of *E. multilocularis*, and thus might help in the design of evidence-based monitoring and control programs. Some recent investigations in the Asian continent were introduced in the symposium with the perspective of featuring distinct types of transmission ecosystems based on intermediate host communities. These can serve as a reference for further in-depth research and help when considering surveillance systems are being considered [23,27,28].

##### *Taenia*

**Figure 1:**
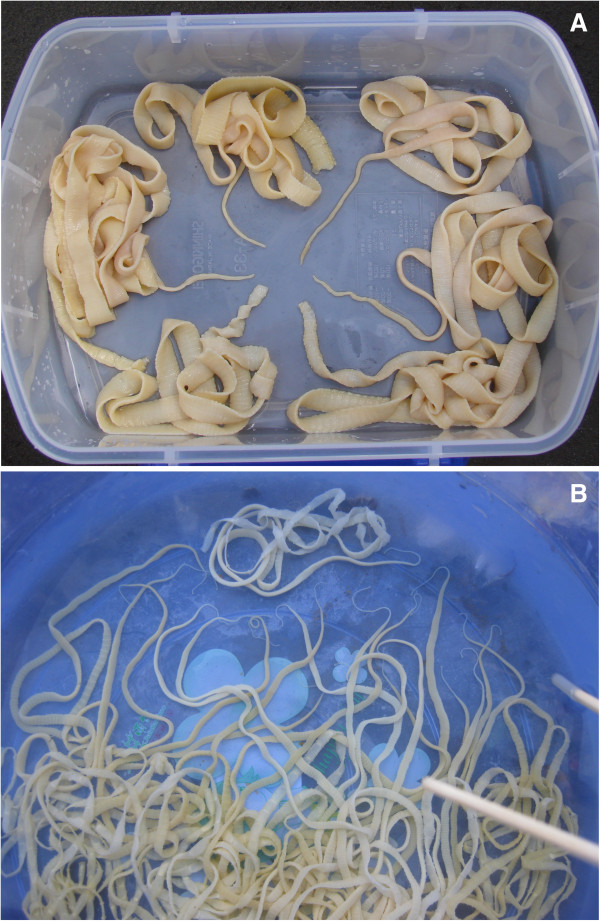
***Taenia *worms collected from a Tibetan agricultural village.***Key:***A**: the worms expelled from a Tibetan girl. **B**: the worms collected from villagers.

Regarding human taeniasis, results from village-based studies in Tibetan communities of southwest China demonstrated that the three human *Taenia* species were co-endemic in farming areas, where neurocysticercosis is an emerging public health concern (see Figure 1). Overall infection rate of taeniasis ranged from 3-20% and seroprevalence of human cysticercosis varied from 4-7% [29,30]. Some participants reported the situation of taeniasis and cysticercosis in humans and livestock in Asian countries, including an infection survey which surprisingly showed that *T. asiatica* was reported in 25 confirmed patients in Japan and linked to the consumption of raw pig liver. However the origins of the taeniasis cluster could not be identified due to a lack of geographical variations in the DNA markers used [31].

#### Control programs and integrated interventions

##### Echinococcosis

Dogs have been recognized as the main definitive hosts of *E. granulosus* (G1 sensu stricto) in China and Central Asian Countries, and an important domestic host of *E. multilocularis*. However, dog-centered hydatid control interventions in rural pastoral communities are more difficult where CE and AE are co-endemic. Many questions need answering such as the natural re-infection rate in dogs for *E. granulosus* and *E. multilocularis,* and the potential for dogs to maintain fox-independent transmission of *E. multilocularis*[23,32]. In this symposium, a one-year observation of seasonal re-infection patterns of dog populations was reported in the Ganzi Tibetan Autonomous Prefecture, Sichuan Province, China, revealing that spring and winter were the seasons when re-infection was most likely to occur. Therefore, a simplified dog deworming scheme, which was both cost-effective and easily implemented, was proposed, in which dog deworming with praziquantel (PZQ) was done mainly in spring and winter [16].

**Figure 2:**
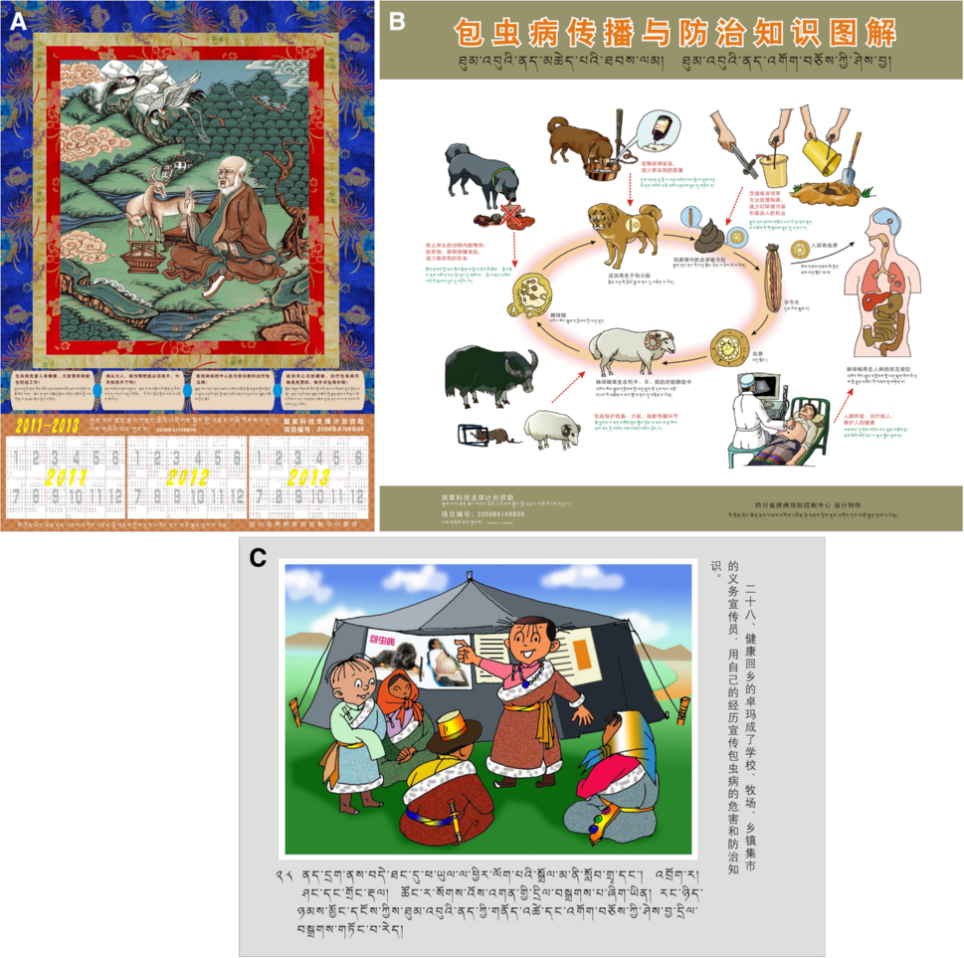
**Designed health educational materials to raise compliance to echinococcosis control activities.***Key:***A**: a Tibetan calendar; **B**: a teaching wall chart; **C**: one page of a picture storybook.

Regarding activities aimed at raising awareness and thus being instrumental in the disease’s control, it was reported that a series of target-specific health educational materials were developed and applied for community-based health education in the Qinghai-Tibet plateau region with the overall aim of raising compliance of Tibetan populations in echinococcosis endemic areas. The materials printed consisted of a Tibetan calendar, a notebook, a pencil bag, a teaching wall chart, a school health textbook, a picture storybook, an animated cartoon and a picture poster, and these were each targeted for the different population needs of religious staff, local government staff, students, and residents (see Figure 2). The assessments for field use showed that these materials are useful in improving knowledge and behavior, and acceptable by most Tibetan populations [33]. Training on diagnosis and treatment of echinococcosis also showed a significant effect on local capacity building in increasing the expertise at prefecture and county levels in Tibetan regions.

Combined use of PZQ and albendazole (ABZ) in a CE patient with multi-infected organs was reported, in which the treatment was a cyclic combination therapy of ABZ at 400 mg twice a day for five days, with PZQ at 800 mg a day for two more days of the week. The regiment was repeated for 10 courses with a two-week interval between courses. The results indicated that both lung and liver cysts reduced in size and no side effects were observed, suggesting a potential additional option for medical therapy [16]. To explore the possibility to increase the bioavailability and cysticidal efficacy of benzimidazoles, the bioavailability of drug-oily solvent suspensions was evaluated. The results demonstrated that the solubility and bioavailability of benzimidazoles in oils may increase to various degrees, and their effects against hydatid cysts were also significantly enhanced [34].

To meet reasonable medical service needs of remote populations from different ethnic groups, a form of teleconsultation was introduced as a novel approach for remote clinical consultation, multi-disciplinary discussion, surgery demonstration, and theoretical training. Echinococcosis patients in remote areas could thus, in theory, benefit from optimal implementation of local treatment, correct referral, as well as high-quality, inexpensive and convenient medical consultation.

##### Taeniasis/cysticercosis

To control *Taenia solium* taeniasis, and consequently the risk of cysticercosis, the usefulness of traditional oral anthelmintic (consisting of pumpkin seeds combined with areca nut extract) was assessed in a community-based treatment for human taeniasis. The results showed that the traditional Chinese herbal treatment was highly effective in expelling intact tapeworms in over 89% of taeniasis cases [29]. Other efforts to reduce transmission have also been made by improving sanitary practices, especially safe disposal of human feces and improved management of pigs. In general, economic development in rural areas of developing countries is also likely to lead to a decline in *T. solium* transmission in the future. Theoretically at least, *T. solium* cysticercosis is capable of being eradicated entirely. More recently, treatment of pigs with oxfendazole and development of an extremely effective vaccine for pigs (Tsol18) have been shown to have great potential in reducing transmission at the community level [35], particularly in the latter, leading to the complete elimination of disease transmission in vaccinated pigs in a field trial in Cameroon [36]. However, many practical issues remain as challenges – including scale and cost – to the implementation of these measures.

#### Molecular biology and diagnostics

##### *Echinococcus*

Based on the need for reappraisal of the species within the genus *Echinococcus* variants, genotypes or strains, some of which are poorly or not infective to humans, a molecular taxonomic discussion sought to reinforce the necessity to revise the taxonomic status and to recognize nine species in the genus *Echinococcus* i.e. *E. granulosus, E. equinus, E. ortleppi, E. canadensis, E. felidis, E. multilocularis, E. shiquicus, E. vogeli,* and *E. oligarthrus*[37-39]. Accumulated molecular evidence provided by participants to the symposium emphasized that, based on the host specificity of *E. canadensis*, the previous genotypes of *E. granulosus* i.e. G6, G7, G8, and G10 should now be regarded as *E. canadensis*[39]. In Russia, *E. canadensis* (G6) was detected in a human infection case. *E. granulosus* was not only found in humans and sheep, but also reported in a cat, while three genotypes of *E. multilocularis* – the Mongolian, Asian, and North-American types – were confirmed from a variety of hosts including the Asian type in human cases [40,41].

Recombinant Em18 from *E. multilocularis* was recommended as a good candidate for the serological detection of almost all active AE cases (PNM classification) [42] and was therefore highly useful for monitoring the progression of AE. Recombinant antigen B8/1 from *E. granulosus* was reported to be another good candidate to detect the majority of active CE cases i.e. CE2, CE3a, and CE3b (WHO-IWGE US classification) [43,44]. Rapid immunochromatographic kits for serodiagnosis of both AE and CE using these recombinant antigens have been developed for field use [45,46]. Also, a genus specific excretory-secretory protein that is overexpressed at the adult stage of *Echinococcus*, EgAgB8/3, was assessed for coproantigen detection in an ELISA test for dog infection, revealing 85% sensitivity and 95.7% specificity [47,48]. Recently, screening and identification of new antigens by immunoproteomic analysis has been conducted by a Chinese group, and 21 antigenic proteins were identified including 12 new ones [49]. A proteomic analysis of the adult stage of *E. granulosus* in dogs was performed and a series of proteins associated with survival, development, movement and modulation were identified, and thus improved the repertoire of candidate proteins for vaccination, immunodiagnosis, and drug development [50]. Vaccine development against experimental rodent alveolar echinococcosis using a novel tetraspanin molecule was introduced in the symposium [51]. To overcome the difficulty in fox sampling, a new quantitative PCR to detect *Echinococcus* species in carnivore feces, which is useful for large-scale as well as fine spatial resolution studies, was presented [16]. Some research reported the study of dynamic changes of cytokines in a mice model infected with *E. granulosus* and showed that Th1 is the major response in the early stage of infection, while Th2 is the major response between the 8^th^ and 16^th^ week after the infection [16].

##### *Taenia*

Molecular phylogeography has highlighted the evolution and dispersal history of zoonotic taeniid cestodes. Based on the phylogeographic analyses for *T. solium* using mitochondrial gene markers, it was reported that there are two genetically distinct geographic subgroups: Asian and African-American [52,53]. Although there has long been a debate as to the specific status of the cestode *Taenia asiatica*, molecular analysis strongly indicates that *T. saginata* and *T. asiatica* were separated into two independent species. On the other hand, hybridization between these two human *Taenia* species seems to be an ongoing event in the co-endemic areas [54-56].

A review of recent advances in immunodiagnosis of cysticercosis and some latest research progress were reported including development of an immunochromatographic test, and a simple and reliable purification method of glycoproteins for immunodiagnosis of human and pig cysticercosis [57]. A reliable serodiagnostic test for human and porcine cysticercosis has been established by a Japanese group [11]. Combination of surveillance tools such as specific serology, copro-detection and molecular epidemiology, in conjunction with GIS and spatial analysis, will assist in understanding the transmission dynamics of *T. solium* between humans and animals, and in optimizing baseline studies and intervention follow-ups and monitoring.

### Gap analysis

It is recognized that substantial gaps exist in the control and research of cestode zoonoses globally, particularly in Asia. Key challenges include capacity building; governance and quality control of research; identifying knowledge gaps of burden estimation of the diseases; development of socioeconomic indicators; resource mobilization; need to develop inter-programmatic, intersectoral prevention and control strategies; and linking research, program and policy for evidence-based decision making.

### Echinococcosis

Epidemiology, ecology and surveillance of *E. granulosus* and CE, and potential for control programs, should include country-wide mapping of cestode zoonoses, and the reporting of medical and veterinary data in a uniform way. As well as that, basic livestock information is required, for example: 1) Do cysts accumulate? 2) Does use of repeated low dose ABZ for G1 nematode infections have any effect on hydatid cysts?

It is necessary to further improve copro-ELISA tests, to assess loop-mediated isothermal amplification (LAMP) and copro-DNA detection, and to run multi-center assessments of copro- and serological tests and their standardization. Up until now, low-cost serological tests for CE and livestock serology remain problematic. In control and prevention of CE, there is a need to: evaluate integrated use of EG95 vaccine and PZQ trials over a five-year period to measure dog re-infection rates in different endemic zones pre- and during PZQ dosing regimes, to minimize dog dosing frequencies that can be used in a given transmission zone/system, to develop setting-specific health education materials, and to further undertake research on experimental dog vaccines. There is also a growing interest in, and importance applied to, systematically analyzing and evaluating the four-five year progress data from dog and human indices for interim assessment of the Chinese National Control Program of Echinococcosis. In the program, mainly comprehensive control measures are taken including dog management and monthly deworming with PZQ, health education, livestock management and immunization, case chemotherapy with ABZ, and surgical treatment.

In epidemiology, ecology and surveillance of AE, the gaps are: mapping distribution; development of landscape risk maps; age structure of small mammal host populations for infection studies; reliable rodent taxonomy for host species in Eurasia; assessment of the DNA profiles in unusual Inner Mongolian *E. multilocularis* isolates (species, genotypes, haplotypes); copro-tests for parasite and host identification; evaluation of uncertainty in diagnostic tests; reliability of serological tests for early AE lesion detection in humans; re-infection rates in dogs; and potential for dogs to maintain a synanthropic cycle without significant fox populations. In control and prevention of AE, the gaps which need to be investigated are centered around: the role of domestic dogs being important in the transmission of *E. multilocularis* in co-endemic areas under hydatid control programs, the problem of stray dogs in some areas, the potential for sustainable dog-small mammal transmission, and the behavior of the red fox, the Tibetan fox and the corsac fox in the wild versus among human communities.

### Taeniasis/cysticercosis

Gaps in research on epidemiology, ecology, and surveillance of taeniasis/cysticercosis need to be focused on distribution maps for countries and accurate hospital data; DALYs calculation for cysticercosis burden, and proportion of epilepsy due to neurocysticercosis in China and the Southeast Asia; accurate porcine cysticercosis data; the minimum effort required to reduce transmission to Ro < 1; transmission dynamics basic parameters for *T. solium*; multi-center assessment of current serological tests (Glycoprotein antigen, Circulating antigen) for human and pig cysticercosis; specificity in relation to *T. hydatigena*; effective DNA detection by LAMP for field use; and increased studies on epidemiology and transmission of *T. solium* in Asia. In control and prevention of taeniasis/cysticercosis, many factors need to be addressed such as the availability of copro-ELISA for mass screening, drugs for taeniasis mass drug administration (PZQ, more restricted niclosamide or others), and integrated use of Tsol18 vaccine and oxfendazole in pig populations through pilot studies.

### Research priorities

Cestode zoonoses, as one group of neglected tropical diseases and neglected zoonotic diseases, mainly affect the poorest sectors of the populations and poor livestock keepers living in rural, often remote and disadvantaged regions of developing countries, and contribute to serious public health problems in the Asian region. China is one of the countries with the highest disease burden of cestode zoonoses in the world, where human CE, AE, and cysticercosis are endemic, and even co-endemic, locally. Human AE is known to be common in certain rural agricultural and pastoral communities. Though globally rare, about 91% of new AE cases globally occurring each year are detected in China with about 380 endemic counties and about 86 million people at risk, particularly in the Qinghai-Tibet plateau region and Western Sichuan. Cestode zoonoses are, however, neglected with little attention from policy-makers, lack of priority within health strategies, and inadequate baseline research.

In this symposium, research priorities were recognized. They included the need to understand the relationship between infectious diseases and poverty, and contribute to priority settings for plans to control those diseases by the introduction of the “one health, one world” concept with trans-disciplinary approaches. It is essential to apply modern tools to obtain scientific evidence on parasite species, strains or isolates, as well as on the confirmation of the diseases themselves, and to further understand epidemiology and transmission ecology. The priorities were also raised to promote optimal opportunities and strategy for control and international collaborations with emphasis on those features, and to improve diagnosis, treatment and control. In addition, estimations of burden of diseases, development of socioeconomic indicators, developing a country-specific control strategy and implementing it, evidence-based interventions, and the use of identified best practices were all identified as important.

## Conclusion

Cestode zoonoses, as a neglected group of potentially life-threatening infectious diseases, pose an important public health challenge globally, particularly in Asian countries. They have substantial socioeconomic impacts as they impact groups beyond those directly affected, disproportionately impact on resource-poor communities in rural or remote areas, and furthermore, impact the health and productivity of livestock.

At this international conference, it was agreed that the following initiatives and recommendations should be undertaken:

•Making a critical assessment on the potential for control of cestode zoonoses focusing on the regions where the populations are at higher risks;

•Establishing a research and validation agenda on new approaches, and novel tools for study and control of the diseases;

•Developing a work-plan of action targeting interventions; and

•Exploiting more resources, favorable public policy, and control options and strategies against cestode zoonoses.

The WHO has recently listed echinococcosis as both a NZD [1] and a NTD [2] to prioritize attention for control strategies leading up to 2018. Cysticercosis/taeniasis has also been placed on the list of the six major helminth diseases of humans for the control and elimination based on a compelling research and development agenda [58,59]. In 2012, the WHO published its roadmap to set out targets for intensified control of selected zoonoses and helminthiases. In January 2013, the WHO published its second report which set milestones for reaching the goals and targets outlined in the roadmap expanding the concept of universal health coverage [60,61].

In this symposium, the possible next steps to achieve an integrated animal-human health approach have been recommended. These include:

•Promoting the concept of “one health” by the development of integrated “control packages” to deal with health problems in people, livestock, and other domestic and wild animals;

•Taking effective measures to raise the profile of the NZDs both internationally and within affected countries;

•Systematically collecting data on the incidence of those zoonoses with support by studies to estimate their dual burden on people and on livestock, to quantify under-reporting, and to identify communities and groups at risk; and

•Investing in the development of new tools needed to effectively control these diseases, particularly in the field of diagnostics.

It is high time to further understand these diseases and fill the gaps on what needs to be done, as well as to make joint efforts to control and research these diseases with a focus on the priority areas identified in this international conference. In addition, it is easy to define the research priorities, but translating these into operational control methods remains a big challenge. This could not be conducted effectively without a good health system and by increasing the prioritization of these diseases.

## Abbreviations

ABZ: Albendazole; DALY: Disability adjusted life years; NTD: Neglected tropical disease; NZD: Neglected zoonotic disease; WHO: World Health Organization; CE: Cystic echinococcosis; AE: Alveolar echinococcosis; PZQ: Praziquantel; MOH: Ministry of Health; NGO: Non-government organization.

## Competing interests

The authors declare that they have no competing interests.

## Authors’ contributions

NX and PSC wrote manuscript; NX, JWY, and WD collected, organized, and reviewed the data; NX, PG, PSC, and AI edited and revised the manuscript; and AI co-organized the symposium. All authors read and approved the final manuscript.

## Authors’ information

NX: Professor, Deputy Director of National Institute of Parasitic Diseases, China CDC; WHO Collaborative Center for Malaria, Schistosomiasis and Filariasis; Key Laboratory of Parasite and Vector Biology, Ministry of Health, 207, Ruijin No. 2 Road, Shanghai 200025, P.R. China;

JWY and WD: Assistant Researchers of the National Institute of Parasitic Diseases, China CDC; WHO Collaborative Center for Malaria, Schistosomiasis and Filariasis; Key Laboratory of Parasite and Vector Biology, Ministry of Health, Shanghai, P.R. China;

PG: Professor of Ecology, Chrono-environment, University of Franche-Comté/CNRS and Institut Universitaire de France, Besançon, France;

PSC: Professor of Biological Sciences and Director of Cestode Zoonoses Research Group, School of Environment and Life Sciences, University of Salford, Salford, Greater Manchester, M5 4WT, UK;

AI: Emeritus and Visiting Professor of Department of Parasitology, Asahikawa Medical University, Midorigaoka Higashi, Asahikawa 078–8510, Hokkaido, Japan.

## Supplementary Material

Additional file 1 Multilingual abstracts in the six official working languages of the United Nations. Click here for file
