# Supplementary material for: Priorities for research and control of cestode zoonoses in Asia
Source: Infect Dis Poverty. 2013 Aug 1;2:16. doi: 10.1186/2049-9957-2-16 (PMC3750256; doi:10.1186/2049-9957-2-16)
Supplement: Additional file 1 — Multilingual abstracts in the six official working languages of the United Nations. [file 2049-9957-2-16-S1.pdf]

Translation of the abstract into the six official working languages of the United Nations

أولويات البحث ومكافحة الأمراض الحيوانية شرطية المنشأ في آسيا

نينغ زيؤوا، جيا ون ياؤوا، وي دينج، باتريك جيرودوو، فيليب كريج، أكيرا إيتوو

الملخــص

على الصعيد العالمي، تُسبب الأمراض الحيوانية شَرطية المنشأ مشاكل خطيرة على الصحة العامة، ولا سيما في آسيا. بين جميع الأمراض الحيوانية المنسية، تُمثل الأمراض الحيوانية ` ŭF Ŏ *cestode zoonoses* أكثر من 75٪ من حالات الإعاقة في العالم خاصة في سنوات العمر الضائعة (DALYs). وقد عُقدت في شنغهاي، الصين، ندوة دولية حول الأمراض الحيوانية شَرطية المنشأ *cestode zoonoses* **تناولت** البحوث ووسائل التحكم وذلك في الفترة ما بين 28 و 30 أكتوبر 2012، من أجل تأسيس جهود مُشتركة لدراسة فعالة وبحث منهجي للتحكم في هذه الأمراض الحيوانية شَرطية المنشأ. وقد شملت الندوة 96 عالماً من منطقة آسيا وخارجها لتبادل الأفكار، وتقديم تقارير عن تقدم البحث، وإجراء تحليل للفجوات، ووضع الأولويات مع التركيز على المنطقة الآسيوية. إن الأهداف الرئيسية لهذه الندوة الدولية هي مُحاولة للاتفاق على حلول للإسراع في إحراز خطوات مُتقدمة نحو تقليل مُعدلات انتقال العدوى، وانخفاض نسبة الوفيات البشرية والحالات المرضية الناجمة عن الأمراض الحيوانية شَرطية المنشأ عبر الثلاث أنواع الرئيسية من *cestode zoonoses* (داء الحويصلات المائية *cystic echinococcosis*، والسنخية المشوكات *alveolar echinococcosis*، وداء الكيسات المذنبة *cysticercosis*)؛ وذلك من أجل تقييم دقيق للإمكانات في التحكم والسيطرة على هذه الأمراض؛ ومن أجل إجراء البحوث وجدول الأعمال المُصادقة على نهج واقعي وجديدٍ، بالإضافة إلى تقديم تقرير عن الأدوات الجديدة للدراسة ولمراقبة الأمراض الحيوانية شَرطية المنشأ _ *cestode zoonoses*.

Translated from English version into Arabic by Manal Abu Nar, through

TRANSLATORS WITHOUT BORDERS

亚洲人兽共患绦虫病研究与防控的优先领域

肖宁，姚嘉雯，丁玮，Patrick Giraudoux, Philip S. Craig, Akira Ito

摘要

人兽共患绦虫病是全球性严重的公共卫生问题，尤其是亚洲深受其害。在所有被忽视的人兽共患病中，人兽共患绦虫病导致的失能调整生命年（DALYs）损失占全球的 75%以上。为了形成合力，探索有效的途径控制人兽共患绦虫病，2012 年 10 月 28 日至 30 日在中国上海举办一次人兽共患绦虫病研究与防控国际研讨会，共有来自亚洲和其它地区的 96 名研究者，卫生管理者，人与动物疾病预防控制专家参加了本次国际研讨会。参会者在会上交流了在人兽共患绦虫病研究和防控方面的进展与经验，进行了差距分析，提炼了研究与防控的优先方向，研讨会尤其关注在亚洲地区开展研究与防控的优先领域。这次国际研讨会就如何加快以下几个方面的进程达成了共识：减少人兽共患绦虫病在人和动物中的传播；降低由囊型棘球蚴病（cystic echinococcosis，CE），泡型棘球蚴病（alveolar echinococcosis，AE）和囊虫病（cysticercosis）这三种主要的人兽共患绦虫病导致人的病死率和发病率；慎重地评估控制这些疾病的潜力和可行性；为目前采用的防控措施和新的方法制订研究和验证计划；报告和使用人兽共患绦虫病研究和控制领域的新成果。

Translated from English version into Chinese by Xiao Ning

Priorités pour la recherche et le contrôle des zoonoses cestodes en Asie

Ning Xiao, Jia-Wen Yao, Wei Ding, Patrick Giraudoux, Philip Craig, Akira Ito

Résumé

Globalement, les zoonoses cestodes peuvent causer de sérieux problèmes de santé, particulièrement en Asie. Parmi toutes les maladies zoonotiques négligées, les zoonoses cestodes comptent pour plus de 75 % des incapacités globales d’ajustement des années de vie perdues (DALY). Un symposium international pour la recherche et le contrôle des zoonoses cestodes a eu lieu à Shanghai, en Chine le 28 au 30 octobre 2012 dernier et l’objectif était d’établir les efforts communs pour étudier et effectuer des recherches sur les approches afin de contrôler ces zoonoses. Ce symposium a réuni plus de 96 scientifiques de l’Asie et d’ailleurs pour échanger sur les idées, présenter des rapports de progression, effectuer une analyse des lacunes et distiller les paramètres de priorité en ciblant particulièrement l’Asie.

Les principaux objectifs de ce symposium international étaient de s’entendre sur des solutions pour accélérer les progrès vers la diminution de la transmission, du taux de mortalité et morbidité causé par trois zoonoses cestodes majeurs (échinococcose cystique, échinococcose alvéolé, cysticercose) et d’évaluer de manière critique le potentiel pour lutter contre ces maladies, d’établir un programme de recherche et de validation de méthodes existantes et nouvelles et de faire un rapport sur de nouveaux outils pour l’étude et le contrôle des zoonoses cestodes.

Translated from English version into French by Johanne Jean-Maître, through

TRANSLATORS
WITHOUT BORDERS

Расставление приоритетов в исследовании и контроле цестодов в азиатском регионе

Нинг Сяо, Цзя-Вен Яо, Вей Динг, Патрик Жироду, Филипп Крейг, Акира Ито

Содержание

По всему миру цестоды вызывают серьезные медицинские проблемы среди населения, и в особенности в азиатском регионе. Среди всевозможных типов зоонозных инфекций в запущенном состоянии, цестод оказывает фатальное влияние на более чем 75% человек и их продолжительность жизни с поправкой на инвалидность. С целью выявления эффективных подходов к контролю зоонозных инфекций 28-30 октября 2012 года в Шанхае был проведен Международный симпозиум по исследованию и лечению цестода. 96 ученых со всей Азии собрались вместе, чтобы обменяться своими достижениями и идеями, провести сравнительный анализ и отобрать приоритетные решения с фокусом на азиатском регионе. В качестве ключевых задач участники симпозиума поставили ускорение процесса по предотвращению передачи зоонозных инфекций и сокращению смертности и распространения болезни, вызванных тремя главными типами цестода (цестозный эхинококкоз, альвеолярный эхинококкоз и цистицеркоз). Они постановили упрочить контроль над заболеванием и начать проверку существующих и инновационных подходов к борьбе с зоонозными инфекциями, а также подбирать новые инструменты исследования и контроля за развитием цестодов.

Translated from English version into Russian by Elanorielle, through

Prioridades en la investigación y control de la zoonosis parasitaria por cestodos en Asia

Ning Xiao, Jia-Wen Yao, Wei Ding, Patrick Giraudoux, Philip Craig, Akira Ito

Resumen

La zoonosis parasitaria por cestodos causa graves problemas de salud pública a escala mundial, especialmente en Asia. De entre todas las enfermedades zoonóticas desatendidas, la zoonosis parasitaria por cestodos supone más del 75% del total mundial de los años de vida ajustados por discapacidad (AVAD) perdidos. Los días 28 al 30 de octubre de 2012 se celebró en Shaghái, China, un simposio internacional sobre la investigación y el control de la zoonosis parasitaria por cestodos, con el objeto de aunar esfuerzos para el estudio de una estrategia eficaz de control de este tipo de zoonosis. El congreso reunió a 96 científicos de la región de Asia y otras zonas para intercambiar ideas, informar sobre los progresos, llevar a cabo un análisis de las deficiencias y extraer las prioridades a establecer, principalmente en la región de Asia. Objetivos clave de este simposio internacional fueron: acordar soluciones para acelerar los progresos encaminados a reducir la transmisión y la morbimortalidad causada por los tres tipos principales de zoonosis parasitarias por cestodos (equinococosis cística, equinococosis alveolar y cisticercosis); evaluar seriamente las posibilidades de controlar estas enfermedades; fijar una agenda de investigación y comprobación de estrategias nuevas y preexistentes y dar a conocer las novedades en métodos de estudio y control de las zoonosis parasitarias por cestodos.

Translated from English version into Spanish by Raquel Bentué, through

TRANSLATORS
WITHOUT BORDERS
